# Metagenomic analysis reveals the abundance changes of bacterial communities and antibiotic resistance genes in the influent and effluent of hospital wastewater

**DOI:** 10.1371/journal.pone.0335723

**Published:** 2025-10-31

**Authors:** Xu Jia, Jiaojiao Peng, Junhong Lv, Yuanting Li, Ziren Luo, Jing Xiang, Yaqin Hou, Qian Zheng, Bin Han

**Affiliations:** 1 Department of Pharmacy, Affiliated Hospital of North Sichuan Medical College, North Sichuan Medical College, Nanchong, China; 2 School of Basic Medical Sciences and Forensic Medicine, North Sichuan Medical College, Nanchong, China; 3 Dazhu County Maternal and Child Health Care and Family Planning Service Center, Dazhou, China; Universidade Catolica Portuguesa, PORTUGAL

## Abstract

The presence of substantial quantities of antibiotics and their metabolites in hospital wastewater can lead to the accumulation of antibiotic-resistant bacteria (ARB) and antibiotic resistance genes (ARGs). Research on the influent and effluent sewage of hospitals is crucial for understanding the effectiveness of wastewater treatment systems in inactivating ARB and ARGs. Key features of microbial communities and ARGs in influent and effluent wastewater – including taxonomic diversity and relative abundance – were assessed via metagenomic sequencing. The treatment process resulted in a reduction of the overall bacterial count in hospital wastewater. However, a notable increase in relative abundance was observed for three phyla, 16 genera, and 21 species post-treatment. Bacteria harboring ARGs were predominantly identified as belonging to *Pseudomonadota* and *Bacillota*. A total of 354 ARGs were detected in the influent, while 331 were identified in the effluent samples, with a general decrease in absolute abundance. Nevertheless, the relative abundance of certain ARGs, such as *mphG*, *fosA8*, and *soxR*, was found to increase in the effluent across all samples. Seasonal fluctuations also played a role in the distribution of microbial communities and ARGs. These findings underscore the role of hospital wastewater treatment systems in reducing the discharge of ARB and ARGs into the environment, while also revealing potential shortcomings in the wastewater treatment process that necessitate further improvement for more effective removal of these ARGs.

## 1 Introduction

Since the discovery of antibiotics in the late 1920s, they have been widely used to treat bacterial infectious diseases [1]. However, the overuse and misuse of antibiotics have significantly contributed to the emergence of antibiotic-resistant bacteria (ARB) and have accelerated the global spread of multidrug-resistant bacteria (MDR) and antibiotic resistance genes (ARGs) [2,3]. This has complicated the task of selecting antibacterial drugs for clinicians and pharmacists and has exerted considerable pressure on the treatment of hospital infections [4,5]. The cure rate for infections caused by superbugs is extremely low, which makes the treatment of clinical microbial infections exceptionally challenging [6]. According to the US Centers for Disease Control and Prevention (CDC), it is projected that by 2050, ARB infections will cause 10 million deaths annually worldwide, surpassing the current number of cancer deaths [7]. Bacteria can acquire resistance to specific antibiotics through gene mutation or through vertical and horizontal transmission. In environments rich in antibiotics, sensitive bacteria are eliminated, allowing resistant bacteria to proliferate. This selective pressure is crucial for the acquisition and accumulation of multiple ARGs [8–10]. Consequently, monitoring and managing antibiotic levels and ARGs in the environment have become critical areas of focus for environmental scientists and the public [11].

In hospital wastewater, the accumulation of ARB and ARGs is a serious problem due to the high levels of antibiotics and their metabolic products [12]. In recent years, studies have found that untreated hospital wastewater in Xinxiang City, Central China, contains various antibiotics, including ofloxacin and cefalexin [13]. The results of bacteriological enumeration show that the sewage of Chittagong Medical College Hospital contains a large number of cefixime-resistant bacteria [14]; similar results have shown high concentrations of ofloxacin in treated hospital wastewater [15]. Tertiary hospitals are advanced referral medical centers providing specialized, high-complexity care, serving as core teaching and research institutions that handle referrals from primary (level I) and secondary (level II) hospitals, which explains their significantly higher Antimicrobial Use Density (AUD) compared to primary care facilities due to treating a greater proportion of critically ill patients with complex infections. Wastewater from tertiary hospitals has been found to contain newer antibiotics, including fourth-generation cephalosporins, cefepime, and meropenem, a carbapenem antibiotic [16]. Antibiotic transformation products (TPs), which arise from metabolism and various other transformations, have been detected in hospital wastewater and sewage treatment plants. Although TPs may exhibit greater environmental persistence, mobility, and aquatic toxicity than parent antibiotics, their potential impacts on microbial communities and ARGs dissemination remain unclear [17]. In recent years, many studies have focused on wastewater treatment plants that receive wastewater from hospitals, communities, and industries. However, research focusing specifically on hospital wastewater treatment processes is still an under-researched area. Conventional disinfection methods, while cost-effective and easy to operate, primarily aim to inactivate microorganisms in wastewater. These methods often overlook the potential persistence of ARB and ARGs. Studies have shown that treated hospital wastewater can still contain opportunistic or pathogenic bacteria, including *Acinetobacter*, *Klebsiella*, *Aeromonas*, and *Pseudomonas.* Meanwhile, many ARGs could not be significantly removed [18]. These resistance genes may enter the aquatic environment through wastewater discharge, posing a potential risk to the ecosystem [19]. Currently, there are no established emission standards for the ARB and ARGs. Analyzing both influent and effluent hospital wastewater is essential for understanding the discharge of clinical ARB and ARGs. It also helps assess the inactivation effects of hospital wastewater treatment systems on these contaminants, which is crucial for understanding the environmental impact of the wastewater treatment process.

The use of antibiotics in Asian hospitals is relatively higher than that in European hospitals [20,21]. In China, per capita antibiotic usage is over five times higher than that in Western countries [22]. Although recent years have seen enhanced management of antibiotic use in major hospitals and large cities in China, the overall trend of misuse has not been fundamentally reversed [23]. The Affiliated Hospital of North Sichuan Medical College, located in Southwest China, is a large national tertiary hospital with comprehensive strength ranking among the top in the province. It serves a population of over 33 million people and has the highest annual number of patients served in Northeast Sichuan. In 2023, the intensity of antibiotic use among discharged patients in this hospital was 36 DDDs (Defined Daily Doses), while the intensive care unit had an antibiotic use intensity of 121 DDDs (Data were obtained from the Pharmacy Department’s quality control briefing at the hospital).

In this study, we used Illumina PE150 sequencing to characterize the bacterial communities and the distribution patterns of ARGs in the hospital’s medical wastewater. By comparing the abundance changes of bacterial communities and ARGs in the influent and effluent, we assessed the role of the hospital’s independent sewage treatment system in reducing the ARGs load in the wastewater. This work provides important insights into both the diversity of ARGs and microorganisms in hospital wastewater and how these may potentially impact the environment receiving hospital effluents. Additionally, our research focuses on the prolonged presence of these bacteria and ARGs following the disinfection treatment of medical wastewater. It highlights the need to enhance existing disinfection infrastructure and underscores the significant environmental risks posed by ARB and ARGs.

## 2 Materials and methods

### 2.1 Hospital wastewater sample collection

The study focused on the medical wastewater of a tertiary general hospital in Nanchong City. Wastewater sample collection was approved in writing by the Affiliated Hospital of North Sichuan Medical College. As samples are environmental and non-human, ethics review was not required. Samples were collected from the influent and effluent of the hospital’s sewage treatment system, which follows the process: sewage pipes → grid well → regulation tank → aeration tank → sedimentation tank → disinfection tank → discharge well → urban sewage treatment plant. Oxone was employed as the disinfectant in the disinfection tank. The sewage treatment system was equipped with a residual chlorine analyzer and an intelligent COD auto-sampler. The residual chlorine level was maintained at 2–8 mg/L (measured at the disinfection tank outlet), while the COD auto-sampler automatically collected and preserved water samples when COD exceeded China’s GB 18466−2005 regulatory limit (250 mg/L), ensuring continuous discharge compliance. Monitoring data corresponding to the sampling months have been incorporated into S4 Table. Samples were collected four times in July 2022 (summer), October 2022 (autumn), December 2022 (winter), and March 2023 (spring). Samples were collected three times from both the influent and effluent at each season. Samples were collected exclusively during dry weather conditions; the influent water samples were taken from the sewage tank prior to the sewage pipes, whereas the effluent water samples were obtained from the discharge well. At each sampling point, a composite sample of approximately 1 L was obtained by combining three ~0.34 L sub-samples collected from different locations. Sample tubes were stored in ice boxes, transported back to the laboratory, and processed within 24 hours. Before collecting the wastewater, the containers were cleaned with methanol, water, and deionized water. From each composite sample, a 0.5 L aliquot was taken for metagenomic sequencing.

### 2.2 DNA extraction and metagenomic sequencing

Hospital wastewater (0.5 L) was centrifuged at 10,000 × g for 30 minutes at 4°C to remove the supernatant. Total DNA from the final sediment was extracted using the TIANamp Soil DNA Kit (DP336, TIANGEN) according to the manufacturer’s instructions. DNA concentration was accurately quantified using a Qubit 2.0 fluorometer. High-quality DNA samples were randomly fragmented to approximately 350 bp. The fragmented DNA underwent end repair, A-tailing, adapter ligation, purification, and PCR amplification to enrich adapter-ligated fragments and complete the library preparation. The libraries were diluted to 2 ng/µl and the insert size was detected using an Agilent 2100. After library construction, sequencing was performed on the Illumina PE150 platform.

### 2.3 Preprocessing of sequencing data and metagenome assembly

The raw data obtained from the Illumina PE150 sequencing platform were preprocessed using Readfq (https://github.com/cjfields/readfq) with the following steps: a) removal of reads containing low-quality bases (quality value <= 38) exceeding a certain proportion (default 40 bp); b) removal of reads with N bases exceeding a certain proportion (default 10 bp); c) removal of reads with adapter overlap exceeding a certain threshold (default 15 bp). Possible human host-derived reads were filtered by alignment with Bowtie2 against the human reference genome (GRCh38/hg38). The clean data were assembled using MEGAHIT assembly software with the parameter –presets meta-large. Scaffolds obtained from assembly were broken from N junctions to obtain N-free sequence fragments called Scaftigs [24–26] (i.e., continuous sequences within scaffolds), and fragments shorter than 500 bp were filtered out. The remaining sequences were used for statistical analysis and subsequent gene prediction.

### 2.4 Gene prediction and abundance analysis

Open reading frames (ORFs) in the scaftigs (≥500 bp) of each sample were predicted using MetaGeneMark (http://topaz.gatech.edu/GeneMark/) and sequences shorter than 100 nt were filtered out. The ORF prediction results were deduplicated using CD-HIT (http://www.bioinformatics.org/cd-hit/). Bowtie2 was used to align the clean data of each sample to the initial gene catalog to calculate the number of reads aligned to each gene. Genes with reads ≤2 in each sample were filtered out to obtain the final gene catalog (unigenes) for subsequent analysis. In the context of metagenomic sequencing, the term “unigenes” commonly denotes the longer, uninterrupted nucleic acid sequences that are constructed during the assembly process from short reads. These sequences embody the full or partial sequences of distinct genes, which is why they are referred to as “unigenes.” The relative abundance of unigenes in each sample was calculated based on the number of aligned reads and gene length using the following formula.


$$Gk=\frac{rk}{\begin{array}{c} Lk \end{array}}•\frac{\text{1}}{\sum_{i=1}^{n}\frac{ri}{Li}}$$

Where *r* is the number of reads aligned to the gene, and *L* is the length of the gene. Based on the abundance information of each gene in the gene catalogue across samples, we conducted basic information statistics, core-pan gene analysis, correlation analysis between samples, and Venn diagram analysis of gene numbers.

### 2.5 Taxonomic analysis of microorganisms in hospital wastewater

DIAMOND software was used to align the unigenes with the sequences of bacteria, fungi, archaea, and viruses extracted from the Micro NR database (Version: 2023.03) (blastp, evalue ≤ 1e-5). For each sequence alignment result, alignments with evalue ≤ the minimum evalue *10 were selected. The LCA algorithm was employed to assign species annotation information to each sequence based on the taxonomic level before the first branching point. Using the LCA annotation results and gene abundance table, the abundance information and gene number table at various taxonomic levels (kingdom, phylum, class, order, family, genus, species) were obtained for each sample. Alpha diversity analysis was based on the results of assembly for species annotation analysis, for which the scaftigs data were used. Shannon: A higher Shannon index indicates greater community diversity. Simpson: The probability that two randomly sampled individuals belong to different species = 1-the probability that two randomly sampled individuals belong to the same species. The greater the Simpson index, the higher the community diversity. Based on the abundance tables at various taxonomic levels, Krona analysis, relative abundance profiling, abundance clustering heatmap generation, and dimensionality reduction (PCA and NMDS) were performed.. ANOSIM analysis was used to test the differences between groups, and Metastats and LEfSe analyses were conducted to identify differential species between groups.

### 2.6 Antibiotic resistance gene diversity in hospital wastewater

Resistance Gene Identifier (RGI) software provided by the CARD database (v3.2.6) was used to align the unigenes with the CARD database (https://card.mcmaster.ca/). RGI uses built-in blastp, and the alignment results were scored using the bitscore value. Based on the RGI alignment results and unigene abundance information, the relative abundance of each ARG was calculated. From the ARGs abundance, bar charts, abundance clustering heatmaps, abundance distribution circle charts, inter-group ARGs difference analysis, and species attribution analysis of ARGs (unigenes annotated to ARGs) were performed. For ARGs with long names, we used abbreviations of their first three words, separated by underscores.

### 2.7 Statistical analysis

Student’s t-test was used to analyze normally distributed data. Non-parametric Kruskal-Wallis rank-sum test and two-sided Wilcoxon rank-sum test were used to analyze non-normally distributed data in RStudio. A p-value < 0.05 was considered statistically significant.

## 3 Results

### 3.1 Illumina sequencing data statistics of hospital wastewater

**Fig 1:**
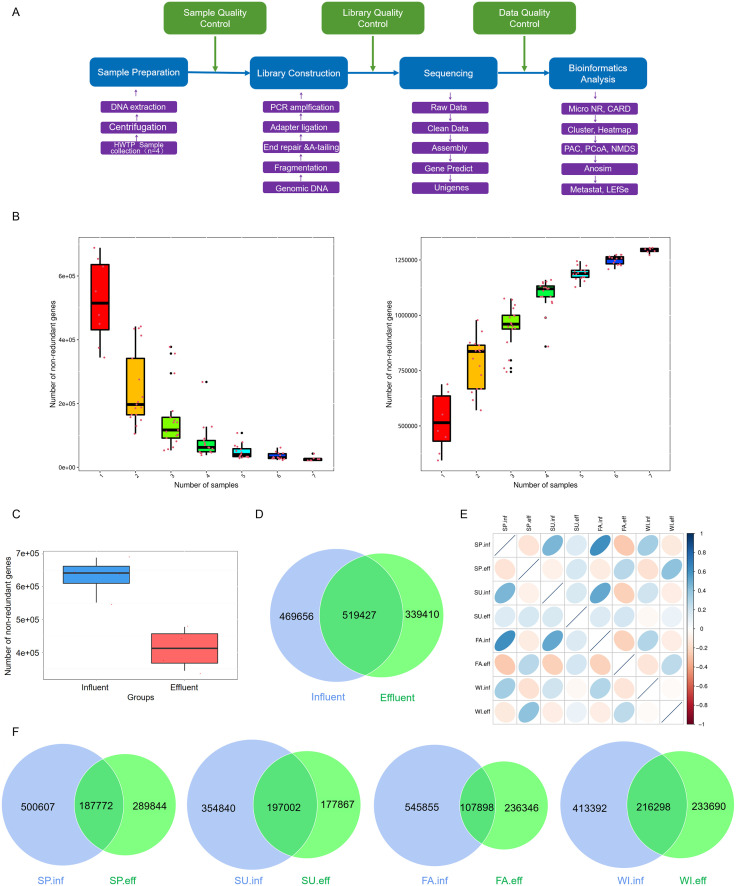
Overview of Metagenomic Sequencing Results. (A) Metagenomics information analysis flowchart. (B) Core gene rarefaction curve (left) and pan gene rarefaction curve (right). (C) Unigenes number differences between groups shown as a boxplot. (D) Venn Diagram of unigenes number differences between groups. (E) Heatmap of sample correlation based on unigenes numbers. Different colors represent the levels of Spearman correlation coefficients; darker colors indicate higher absolute correlation coefficients. Ellipses leaning to the right indicate positive correlation, while those leaning to the left indicate negative correlation. The flatter the ellipse, the higher the absolute value of the correlation coefficient. (F) Venn Diagram of unigenes numbers in water samples from different seasons, showing both influent and effluent samples.

A total of 353,541,340 reads were obtained from 8 samples, with an average of 44,192,667 reads per sample, and the read count differences between samples were small. After removing low-quality reads, the proportion of high-quality reads was approximately 99.72% on average. The sequencing data output and preprocessing results are shown in S1 Table. The base quality and distribution of sequencing are shown in S1 Fig., with the quality of sequencing data for all samples mainly distributed above Q20. All high-quality reads were assembled into 1,358,363 Scaftigs (>500 bp), with an average of 169,795 per sample (S2 Table), and the length distribution of each sample was shown in S2 Fig. Starting from the Scaftigs of each sample, a total of 2,421,435 ORFs were predicted (S3 Table), and after removing redundancy, the final gene catalogue (unigenes) used for analysis was obtained, with the length distribution shown in S3 Fig. The metagenomics information analysis flow was shown in Fig 1A.

### 3.2 Unigenes number and sample correlation analysis of influent and effluent water samples in hospital wastewater treatment system

Given that metagenomic samples typically include a blend of genomes from multiple microorganisms, high-throughput sequencing techniques are employed to acquire a plethora of short sequence reads. Subsequently, bioinformatics tools are utilized to assemble these reads into unigenes, allowing for further examination of the genetic diversity and functional capabilities present in the sample. Starting from the unigenes abundance in each sample, the unigenes number information of each sample can be obtained. By randomly sampling different numbers of samples, core and pan gene rarefaction curves were constructed (Fig 1B). The results show that the number of core genes (genes present in all analyzed metagenomes) decreases when more sample metagenomes are added to the analysis, while the number of pan genes (the total gene set observed across all metagenomes) continues to expand after adding 7 metagenomes. This approach, adapted from traditional genomic analyses to our metagenomic data, provides insights into the shared and unique genetic content across our environmental samples. The comparison results of unigenes numbers between groups show that the unigenes number in the metagenome in the effluent of the hospital wastewater treatment system is significantly lower than that in the influent (Fig 1C). This difference suggests a potential impact of the treatment process on the microbial community composition and genetic diversity. In order to examine the distribution of gene numbers among specific samples or groups, and to analyze the shared and unique gene information between different samples, Venn diagrams of the differences in unigenes numbers between samples or groups were drawn. A total of 989,083 unigenes were obtained in the influent, and 858,837 unigenes were obtained in the effluent. The Venn diagram results show that there are 519,427 overlapping unigenes between the influent and effluent (Fig 1D). The number of unigenes obtained in spring and autumn is higher than that in summer and winter, and there is a certain overlap of unigenes between the influent and effluent in different seasons. The largest overlap of 216,298 unigenes occurs in winter (Fig 1F). The correlation results of unigenes abundance between samples show that the correlation coefficients between influents and between effluents in different seasons were positive, while the correlation coefficients between the influent and effluent were negative (Fig 1E).

### 3.3 The impact of hospital wastewater treatment systems on the microbial composition and relative abundance

**Fig 2:**
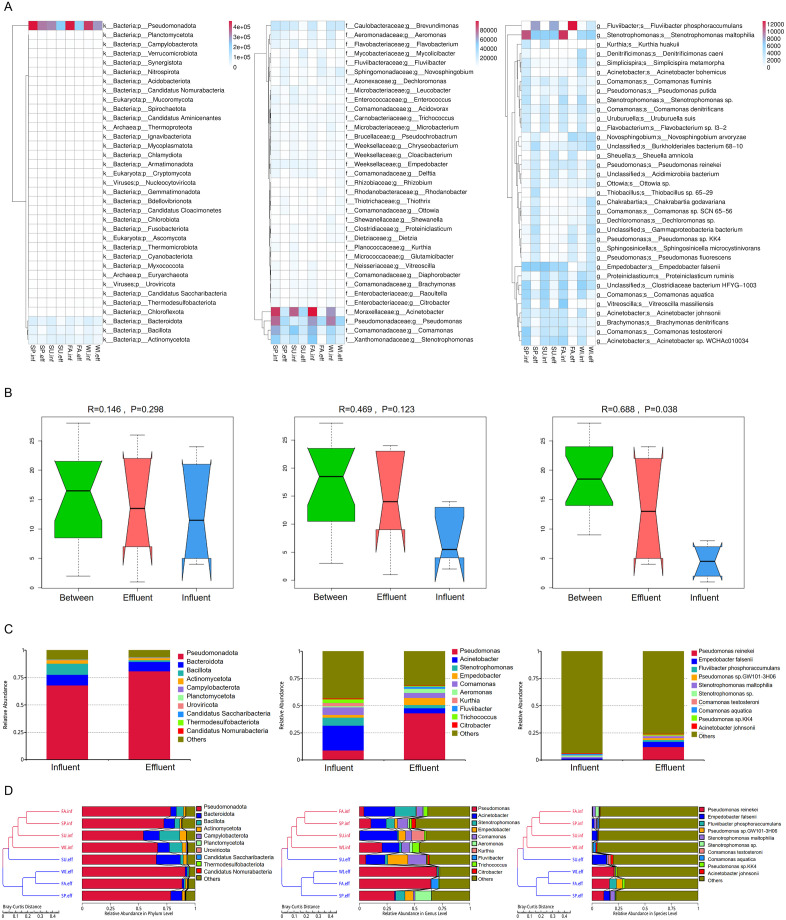
Community Structure and Diversity of the Microbiota from Hospital Wastewater. (A) Heatmap of the numbers of unigenes annotated to the phylum (left), genera (middle), and species (right) levels in all samples. Different colors represent the levels of unigenes numbers. (B) ANOSIM boxplot ranks between and within the influent and effluent groups at the phylum (left), genera (middle), and species (right) levels. (C) Stacked barplot of the relative abundance at phylum (left), genera (middle), and species (right) levels for taxonomic profiling of bacterial communities. Labels represent the top 10. Samples are grouped by influent and effluent. (D) Sample clustering based on Bray-Curtis Distance and relative abundance at the phylum (left), genera (middle), and species (right) levels in all samples.

Monitoring data from hospital wastewater (S4 Table) revealed that both COD and residual chlorine levels during the sampling months complied with discharge standards. Additionally, the effluent water temperature exhibited marked seasonal variations, with the largest observed temperature difference reaching approximately 17°C. To gain a comprehensive understanding of the changes in microbial composition, we analyzed the variations in microbial abundance in both the influent and effluent water samples at the phylum, class, and genus levels. Krona analysis was used to display the relative abundance of microorganisms at the kingdom, phylum, and class taxonomic levels. The results showed that bacteria accounted for the largest proportion both in the influent and effluent water, approximately 95%–97%, with no significant difference in proportion between the influent and effluent. The proportion of fungi, DNA viruses, and archaea varies greatly between samples. Our DNA-based metagenomic approach and sample processing protocol (10,000 × g centrifugation without electronegative membrane filtration) were not optimized for broad viral recovery, thus, our results likely underestimate true viral diversity by missing RNA viruses, ssDNA viruses, and free viral particles. At the phylum level, *Pseudomonadota* dominates in both influent and effluent samples, with its proportion ranging from 54% to 91%. At the class level, *Gammaproteobacteria* was the most abundant group, present in proportions that vary between 41% and 77%. Compared to the influent, the proportion of *Pseudomonadota* and *Gammaproteobacteria* increases in the effluent (S4 Fig). Given the relatively low abundance of other microorganisms, further analysis primarily focuses on the composition of bacteria at the phylum, genus, and species levels. We analyzed the alpha diversity indices (Shannon, Simpson) of different samples at both the phylum and genus levels. Results demonstrated that the Shannon and Simpson indices of the effluent water samples were lower than those of the influent water samples across all four seasons, observed at both taxonomic levels. Notably, at the phylum level, the Simpson index of the effluent water samples was higher than those of the influent only during summer (S5 Table). Starting from the numbers of unigenes annotated to different taxonomic levels, we selected the top 35 bacteria for visualization. Compared to the influent, the numbers of unigenes annotated to the phylum and genus levels in the effluent show a significant decreasing trend. At the species level, although the numbers of unigenes annotated to most species decreased in the effluent, the numbers of unigenes annotated to some species show an increasing trend, such as *Fluviibacter phosphoraccumulans*, *Pseudomonas reinekei*, *Acidimicrobiia bacterium*, and *Ottowia sp.* (Fig 2A). The ANOSIM analysis results based on relative abundance show that the R-value at the phylum, genus, and species levels are all greater than 0, indicating that the between-group differences are greater than the within-group differences, but only the between-group differences at the species level are statistically significant (Fig 2B). Starting with relative abundance, we analyzed the top 10 bacteria selected from different taxonomic ranks. At the phylum level, the top 10 are *Pseudomonadota, Bacteroidota, Bacillota, Actinomycetota, Campylobacterota, Planctomycetota, Uroviricota, Candidatus Saccharibacteria, Thermodesulfobacteriota,* and *Candidatus Nomurabacteria*. At the genus level, the top 10 are *Pseudomonas, Acinetobacter, Stenotrophomonas, Empedobacter, Comamonas, Aeromonas, Kurthia, Fluviibacter, Trichococcus*, and *Citrobacter*. At the species level, the top 10 are *Pseudomonas reinekei, Empedobacter falsenii, Fluviibacter phosphoraccumulans, Pseudomonas sp. GW101-3H06, Stenotrophomonas maltophilia, Stenotrophomonas sp., Comamonas testosteroni, Comamonas aquatica, Pseudomonas sp. KK4,* and *Acinetobacter johnsonii* (Fig 2C). The distribution of relative abundance in each sample at the phylum, genus, and species levels is consistent with the overall situation. The Bray-Curtis distance matrix analysis based on relative abundance shows that at the phylum, genus, and species levels, all influent samples cluster together, while all effluent samples also form a distinct cluster. This indicates that the treatment process is the dominant factor shaping microbial community composition. Notably, influent communities vary considerably across seasons—for example, SU.inf and WI.inf are distantly separated on the dendrogram. Although the effluent communities are consistently dominated by disinfectant-resistant bacteria, the relative abundance of dominant taxa varies seasonally. *Pseudomonas reinekei* was identified as the most prevalent survivor and the absolute dominant bacterium in the water following Oxone disinfection. However, the effluent community in summer exhibits slightly greater diversity (Fig 2D). These findings suggest that seasonal variations—likely mediated through changes in inlet water quality, temperature, or other physicochemical conditions—provide a subtle yet discernible niche for secondary resistant bacteria, thereby indirectly modulating the specific structure of the effluent microbial community. Starting from the relative abundance across various taxonomic ranks, we analyzed the changes in the top 35 bacteria in the influent and effluent water samples by creating a heatmap for visualization. Compared to the influent samples, the effluent samples have 21, 17, and 10 types of bacteria with increased relative abundance at the phylum, species, and genus levels, respectively. As shown in S5D Fig. The PCA, PCOA, and NMDS analyses results are consistent with the Bray-Curtis distance matrix analysis results (S5A–C Fig).

### 3.4 Metastats and LEfSe analysis of inter-group differences in bacteria

**Fig 3:**
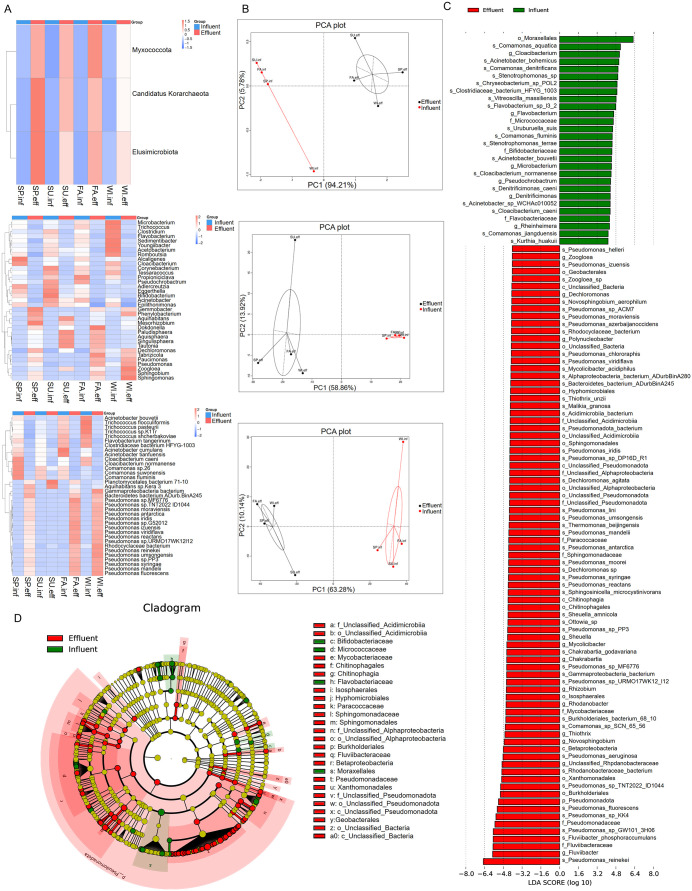
Analysis of Significantly Different Bacteria. (A, B) Heatmap and Principal Component Analysis (PCA) of significantly different bacteria abundances at various taxonomic levels in all samples: phylum (top), genera (middle), and species (bottom). In (A), the left side shows the clustering tree, and the middle heatmap displays the Z-scores of relative abundances after standardization. In (B), the x-axis represents the first principal component, with the percentage indicating its contribution to sample differences; the y-axis represents the second principal component, with the percentage indicating its contribution to sample differences. (C, D) LDA integrated with effect size (LEfSe), comparing influent vs. effluent. (C) shows the differences in bacteria abundance (all p < 0.05, shown only when the absolute value of the LDA Score is greater than 4). (D) displays the phylogenetic distribution of bacteria in a cladogram.

To study the significantly different bacteria in various taxonomic ranks between groups, the Metastats method was used to perform hypothesis testing on relative abundance between groups, and bacteria with significant differences (q < 0.05) were screened out. The clustering heatmap of significantly different bacteria abundances is shown in Fig 3A. At the phylum level, the abundance of *Myxococcota*, *Candidatus Korarchaeota,* and *Elusimicrobiota* increased significantly in the effluent. At the genus level, 19 genera showed a significant decrease and 16 genera showed a significant increase in the effluent compared to the influent. At the species level, 14 species show a significant decrease and 21 species show a significant increase in the effluent compared to the influent. The PCA analysis of significantly different bacteria at the phylum, genus, and species levels showed the differences between influent and effluent samples (Fig 3B). To further screen for biomarker bacteria with significant differences between groups, the Wilcoxon rank-sum test was used to detect different bacteria between groups, and the LDA analysis to evaluate the effect size of different bacteria. The LDA bar chart results showed that there are 28 different bacteria with LDA Scores greater than 4 in the influent, with the top 3 contributing bacteria being *Moraxellales, Comamonas aquatica,* and *Cloacibacterium*. In the effluent, there are 83 different bacteria with LDA Scores greater than 4, with the top 3 contributing bacteria being *Fluviibacteraceae, Fluviibacter,* and *Pseudomonas reinekei* (Fig 3C). The evolutionary branch diagram of different bacteria shows the important microbial groups from the phyla to the genera level in the influent (green) and effluent (red), with the different bacteria mainly concentrated in *Pseudomonadota, Betaproteobacteria, Burkholderiales,* and *Pseudomonadaceae.*

### 3.5 Overview of ARGs distribution in the influent and effluent of hospital wastewater

**Fig 4:**
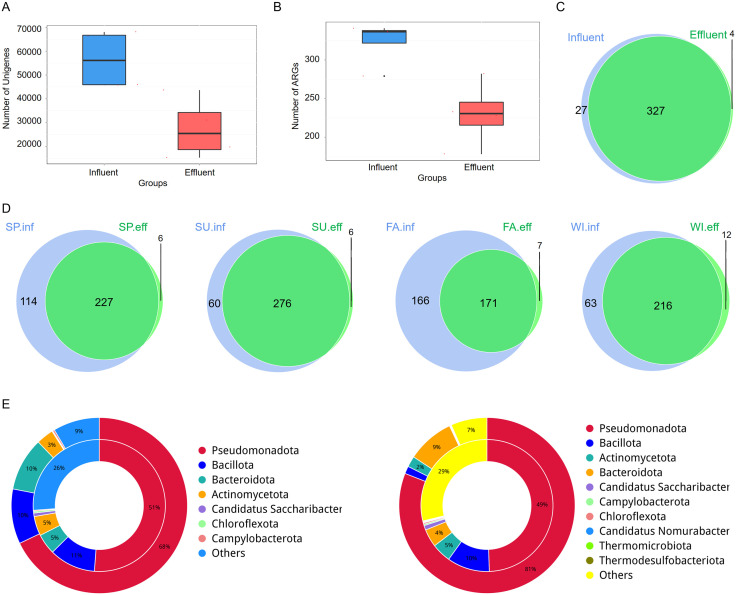
Overview of ARGs Annotation Results. (A) The number of unigenes annotated to ARGs between groups. (B) Boxplot of ARGs number differences between groups. (C) Venn Diagram of ARGs number differences between groups. (D) Venn Diagram of ARGs numbers in water samples from different seasons, showing both influent and effluent samples. (E) Dual-circle plot of ARGs phyla attribution in influent (left) and effluent (right) samples. The inner circle shows phyla distribution for each ARG type, and the outer circle shows phyla distribution for all samples within the group.

**Fig 5:**
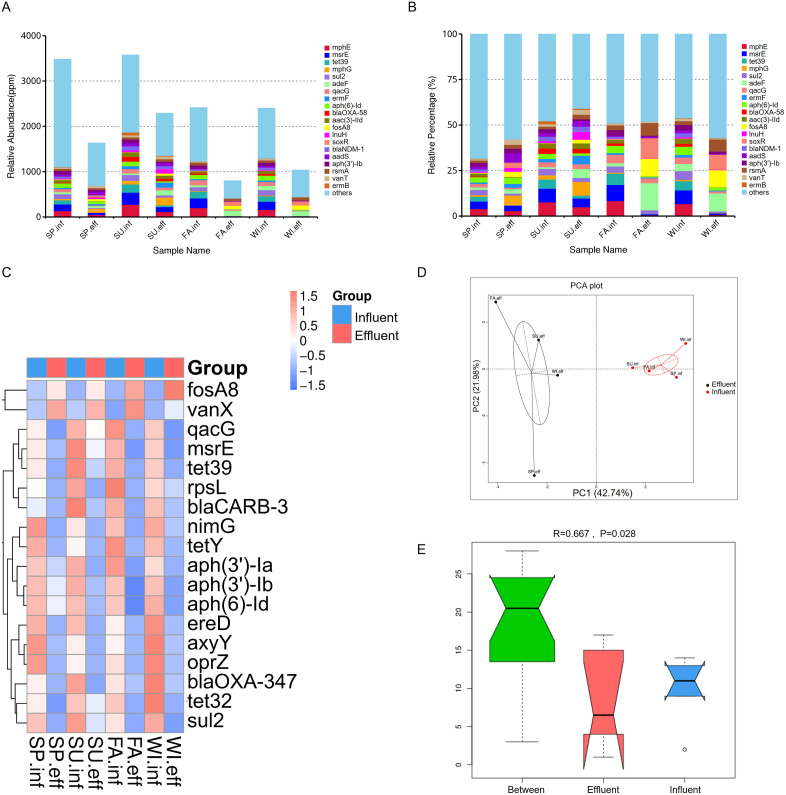
Relative Abundance and Diversity of ARGs. Based on RGI alignment results combined with unigene abundance information, the relative abundance of each ARG was calculated. (A) The relative abundance and (B) percentage of the top 20 most abundant ARGs in each sample. (A) shows the results with the original relative abundance data magnified by 10^6. The relative abundances and percentages of all other ARGs not in the top 20 are grouped as “other.” (C, D) Heatmap and PCA of significantly different ARGs abundances. (C) shows the clustering tree on the left, with the middle heatmap displaying the Z-scores of relative abundances after standardization. In (D), the x-axis represents the first principal component, with the percentage indicating its contribution to sample differences; the y-axis represents the second principal component, with the percentage indicating its contribution to sample differences. (E) ANOSIM boxplot ranks between and within the influent and effluent groups based on ARGs relative abundance.

Widespread ARGs, coupled with the abuse of antibiotics have led to irreversible changes in the microbial communities of the human body and environment. Microorganisms that carry ARGs have a survival advantage in the presence of antibiotics, leading to an increase in their numbers. These changes to the microbial communities can be long-lasting or permanent, as once resistance has developed, it can spread through microbial populations and persist even after the selective pressure (antibiotic use) is removed. The unigenes obtained in the early stage were compared with the CARD database, with an average of 56,515 unigenes annotated to about 323 ARGs in the influent samples. In the effluent, an average of 27,440 unigenes were annotated to about 230 ARGs (Fig 4A, 4B). The difference in the number of ARGs obtained between the influent and effluent in autumn is relatively large, but overall, there is a significant overlap in the ARGs enriched in the influent and effluent, with only a few unique to the effluent. *catB, folC, rbpA, and fosXCC* were detected only in the effluent (Fig 4C, 4D). ARG-host associations were inferred based on the taxonomic annotation of contigs containing ARGs. Only ARGs on contigs ≥ 1000 bp with consistent taxonomic assignments (≥ 80% agreement) were included in the phylogenetic attribution analysis, representing approximately 65% of detected ARGs. The phyla information corresponding to each ARG in the samples within the group can be obtained, as shown in Fig 4E. The inner circle shows the phyla attribution of ARGs, and the outer circle shows the phyla distribution of all samples in the group. The Detected ARGs in the influent were predominantly sourced from *Pseudomonadota* (51%) and *Bacillota* (11%), and the effluent was similar to influent, mainly attributed to *Pseudomonadota* (49%) and *Bacillota* (10%) (Fig 4E). From the relative abundance of ARGs, the content and percentage of ARGs in each sample were calculated, and the top 20 ARGs were *mphE, msrE, tetA39, mphG, sul2, adeF, qacG, ermF, aph(6)-Id, blaOXA-58, aac3-lld, fosA8, lnuH, soxR, blaNDM-1, aadS, aph(3’)-IIb, rsmA, vanT, and ermB.* Overall relative abundance of ARGs showed a decreasing trend in the effluent compared to the influent (Fig 5A), whereas the proportion of a certain gene did not vary consistently between the influent and effluent (Fig 5B).

### 3.6 Analysis of significantly different ARGs in the influent and effluent of medical wastewater and distribution of resistance mechanisms

**Fig 6:**
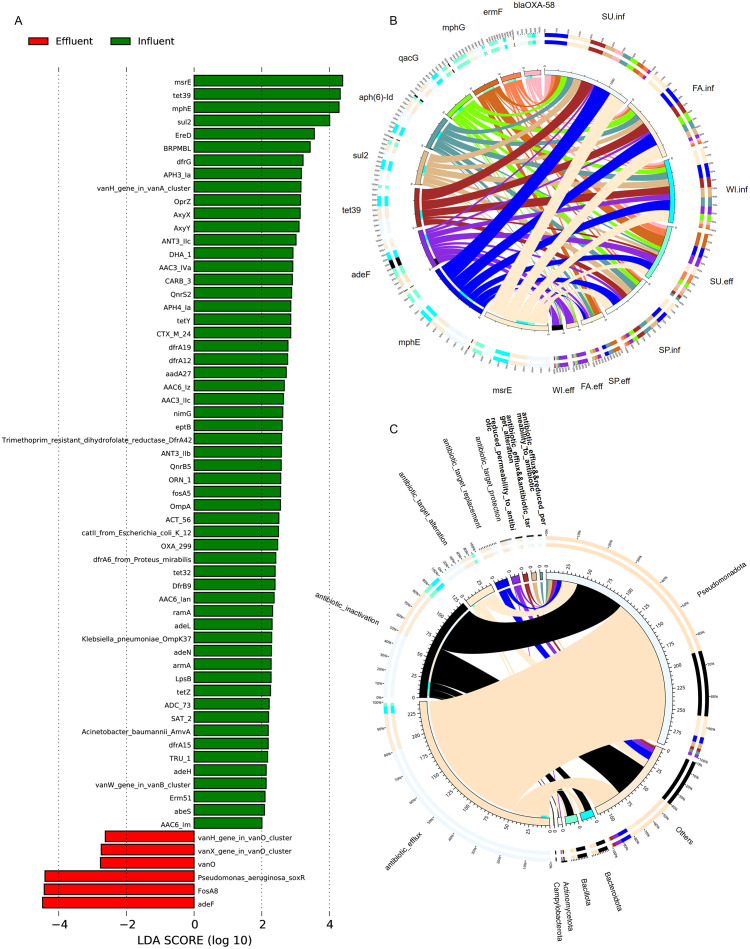
Analysis of Significantly Different ARGs and Resistance Mechanisms. (A) LEfSe analysis identified the most differentially abundant ARGs between influent and effluent groups (all p < 0.05, shown only when the absolute value is greater than 2). (B) Overview circle plot of the top 10 ARGs with the highest abundance. The circle plot is divided into two parts: the inner circle shows different samples and ARGs in different colors, with the scale representing relative abundance in ppm. The left side shows the total relative abundance of each ARG in all samples, while the right side shows the total relative abundance of each ARG in different samples. The outer circle shows the relative percentage of each ARG within the group on the left side, and the relative percentage of each ARG in different samples on the right side. (C) Overview circle plot of resistance mechanisms and phyla. The inner circle shows different phyla and resistance mechanisms in different colors, with the scale representing gene numbers. the left side shows the total number of ARGs containing that resistance mechanism within the phyla, and the right side shows the total number of ARGs that the phyla contains within different resistance mechanisms; The outer circle shows the relative proportion of ARGs in each phyla with the resistance mechanism on the left side, and the relative proportion of ARGs in each resistance mechanism within the phyla on the right side.

To study the significantly different ARGs between groups, Metastats and LEfSe analyses were used to assess the impact of different ARGs. The heatmap of significantly different gene abundances is shown in Fig 5C. Compared to the influent, there were 16 significantly decreased ARGs and 2 significantly increased ARGs in the effluent, namely *fosA8* and *vanX*. PCA and ANOSIM analyses showed that the differences between groups were greater than those within groups (Fig 5D). The LDA bar chart results indicated that there were 57 different ARGs with an LDA Scores greater than 2 in the influent, with the top three contributing ARGs being *msrE*, *tetA39,* and *mphE*. In the effluent, there were 6 different ARGs with an LDA Score greater than 2, with the top three contributing ARGs being *soxR, fosA8,* and *adeF* (Fig 6A).To visually observe the abundance ratio of ARGs in each sample, we drew an overview circle plot was drawn for the top 10 ARGs with the highest abundance. The results showed that the abundance of ARGs in the influent was higher than that in the effluent. However, the abundance of ARGs in the effluent in summer was higher than that in the influent in spring, and the abundance of ARGs in the influent in summer was higher than that in the influent in other seasons. This suggests that seasons may affect the distribution of ARGs in water. The analysis of the top 10 ARGs revealed that *msrE* and *mphE* exhibited the highest abundance in the influent. Additionally, *mphG* displayed the highest abundance in the effluent during spring and summer, while *adeF* exhibited the highest abundance in the effluent during autumn and winter (Fig 6B). Finally, by classifying the resistance mechanisms of ARGs and analyzing the relationship between resistance mechanisms and phyla, the results showed that the resistance mechanisms mainly included antibiotic efflux, antibiotic inactivation, and antibiotic target alteration. These resistance genes were mainly attributed to *Pseudomonadota* (Fig 6C).

## 4 Discussion

Hospital wastewater merits particular attention as a significant source of environmental resistance dissemination due to its high abundance of ARGs. Carbapenemase gene abundance in hospital wastewater is significantly higher in Asia than in Europe [27], yet European effluents persistently release ARGs into the environment [28]. Even in low-resistance countries like Norway, hospital wastewater harbors 1,205 ARGs, including novel metallo-β-lactamase genes, with 96 metagenome-assembled genomes (MAGs) taxonomically matching those in wastewater treatment plants (WWTPs) [29]. ARG abundance and diversity exhibit substantial spatiotemporal heterogeneity. Nationwide metagenomic sequencing of hospital effluents in Wales revealed that the smallest facility (Aberystwyth Hospital) demonstrated the highest ARG load—likely attributable to more effective antibiotic stewardship and waste management practices in larger hospitals [27]. Specialized infectious disease hospitals show significantly higher sulfonamide ARG abundance than general hospitals, reflecting intensive sulfa drug usage for infections like Pneumocystis pneumonia in immunocompromised patients [30,31]. COVID-19 disinfectant overuse intensified co-occurrence of tetracycline resistance gene *tetX* and extended-spectrum β-lactamase gene *bla*_*NDM*_, accelerating multidrug-resistant pathogen emergence [32]. Municipal WWTPs display seasonal variations in ARG profiles, such as elevated clinically critical ARGs in winter/spring in Beijing, accompanied by concurrent changes in bacterial community structure and ARG diversity [33]. Therefore, systematic characterization of ARGs in hospital wastewater treatment systems’ influents and effluents holds significant scientific value. In our current study, we employed metagenomic techniques to reveal the diversity and abundance of microorganisms and ARGs in water samples from the wastewater treatment system of a general hospital. By comparing the seasonal variations in microbial abundances and ARG abundances in the influent and effluent, we aim to decipher regional resistance profiles and to quantify impacts of seasonal variations and disinfection processes on the resistome.

The total length of scaftigs, the number of ORFs, and the number of unigenes identified from the assembly of influent water samples were significantly higher than those of effluent samples, while there was no significant difference in the clean data yield. The total length of scaftigs reflects the overall genetic information within the metagenome; the number of ORFs indicates the potential protein-coding capacity of the microbial community, and the number of unigenes reveals the richness of known functional genes [34]. Dias MF et al. demonstrated that a modified activated sludge system was effective in reducing both the investigated pathogen’s proportion and ARB concentration [35]. These metric changes between influent and effluent samples indirectly indicate that the treatment process has reduced the genetic diversity of the microbial community to some extent. The number of unigenes in samples from both sampling points showed seasonal variations. The number of unigenes in influent samples was lower in summer compared to the other three seasons. Notably, even though the number of unigenes in influent samples was lower in winter than in autumn, the number of unigenes in effluent samples was higher in winter than in autumn. This suggested that the treatment process might be less effective in winter, potentially due to factors such as lower temperatures affecting the disinfection process. The primary disinfectant in the hospital sewage treatment system for this study was Oxone. When dissolved in water, Oxone releases various active components such as active oxygen and hydroxyl radicals, which have broad-spectrum microbial killing effects and can inactivate chlorine-resistant bacteria in water and pipe networks [36,37]. According to meteorological data from China, the study area is characterized by a subtropical monsoon climate with distinct seasonal temperature variations. Winters are relatively cold, with average temperatures ranging from 6 to 8°C. Previous studies have indicated that the disinfection efficiency of Oxone is temperature-dependent within the range of 10–30°C, with its bactericidal efficacy improving as temperature increases. Given that lower temperatures can attenuate the activity of Oxone, we speculate that the higher number of unigenes observed in effluent samples during winter may be attributed to reduced Oxone performance under cold conditions [38].

We analyzed the microbial community at the phylum, genus, and species taxonomic levels. Comparing the biological communities across different seasonal influent and effluent sampling points, our results showed significant differences in the overall community distribution at the species level, with no significant differences observed at the phylum and genus levels. A study incorporating metagenomic datasets from Chinese hospital wastewater between 2018 and 2022 revealed that *Pseudomonadota* consistently serves as a primary carrier of ARGs at the phylum level, yet there are significant regional variations in the genetic background of ARGs at the genus and species levels [39]. In the influent and effluent wastewater from an eye specialty hospital and a general hospital, *Pseudomonadota* similarly dominates [30,31]. Consistent with these findings, we found that *Pseudomonadota* constitutes over 60% of the microbial community at both the influent and effluent of the wastewater treatment system, with the majority of ARGs being attributed to this phylum. When compared to findings from different types of hospitals in other regions, our results reaffirm the presence of regional and hospital category-specific differences in the distribution of microorganisms at the genus and species levels. These findings are consistent with the microbial distribution observed in non-hospital healthcare facilities and municipal sewage [40]. The consistently lower Shannon and Simpson indices in effluent vs. influent across seasons and taxonomic levels indicated that the treatment process significantly reduces microbial community richness and/or evenness. This general reduction in diversity serves as an indicator of the treatment process’s effectiveness in removing or inactivating diverse microorganisms. However, the summer anomaly at the phylum level revealed a contrasting pattern: a higher Simpson index in effluent versus influent signified increased diversity within the effluent community. This deviation highlights the impact of seasonal conditions (e.g., elevated water temperature, altered treatment efficiency). At the phylum, genus, and species levels, our results indicated a significant decrease in the relative abundance of most bacterial communities from influent to effluent, but a significant increase in that of some bacterial communities— especially *Pseudomonadota*, *Pseudomonas,* and *Pseudomonas reinekei*. The microbial community evenness and richness in the wastewater significantly changed after treatment, which could be attributed to different treatment methods and the formation of biofilms. Contrary to our research findings, activated sludge wastewater treatment may enrich *Actinobacteria*, while direct chlorination disinfection processes may increase the relative abundance of *Bacteroidetes.* Wastewater microbial communities are shaped by watershed type and treatment methods. Activated sludge treatment cultivates diverse microbes to break down pollutants, transforming the community from unstable and pollution-tolerant to a diverse, stable, and efficient artificial ecosystem—thereby increasing bacterial diversity and richness. In contrast, disinfection methods like UV or chemical treatments remove pathogens through biocidal or selective pressure, ensuring biological safety. As a result, Oxone treatment typically reduces microbial diversity [31,35,41]. Many studies suggest a close correlation between biofilm formation and resistance of wastewater isolates; *Pseudomonas aeruginosa* and *Escherichia coli,* known for their biofilm-forming potential, persist long-term in the wastewater system [42]. Notably, the community distribution in summer effluent samples differed from other seasons, including the dominance and abundance of bacterial communities. Rising summer temperatures, with water temperatures around 25–30°C, may accelerate bacterial reproduction, making the bacterial community structure in summer effluent samples more complex than in other seasons. Luo F’s research also confirmed that the microbial communities in summer water samples were primarily affected by temperature rather than other environmental parameters, while those in winter water samples were significantly correlated with COD, DO, and turbidity [43]. However, given the many possible reasons for the differences in microbial community structure, more comprehensive and in-depth research is needed to verify this hypothesis.

Our study showed a general decrease in the number of ARGs after treatment by the sewage treatment system, with ARGs varying by season. The number of ARGs decreased significantly in spring and autumn, while the treatment was less effective in eliminating ARGs during summer and winter. Notably, unlike the number of unigenes annotated to microbial communities, the number of unigenes annotated to ARGs in summer effluent samples was higher than in other seasons. Studies have shown that high environmental temperatures could enhance gene expression, induce gene mutations, and help maintain antibiotic resistance [44], leading us to hypothesize that the higher temperatures of summer may lead to an increase in ARGs in effluent samples. The ARGs identified in both influent and effluent samples were generally consistent in their background, with over 50% affiliated with *Pseudomonadota* and *Bacillota*, aligning with their known dominance in the human gut microbiota [45]. Our study showed that the relative abundance of ARGs in effluent samples generally decreased compared to influent samples, although not entirely eliminated, and the dominant ARGs varied between influent and effluent samples. The dominant ARGs in influent samples were consistent, mainly *msrE* (macrolide efflux pump gene) and *mphE* (macrolide phosphotransferase gene), while the dominant ARGs in effluent samples showed seasonal variations. During autumn and winter, *adeF* (multidrug efflux pump gene) dominates the effluent profile. Critically, the enrichment of adeF-mediated multidrug resistance in effluent warrants heightened vigilance, as it may foster “superbugs” resilient to conventional water treatment, posing dual threats to aquatic ecosystems and public health. Conversely, *mphG* (a distinct macrolide phosphotransferase gene) exhibits peak effluent abundance in spring and summer; its dissemination could exacerbate clinical failure risks for macrolide antibiotics (e.g., azithromycin). Consequently, our findings underscore a key operational implication: Seasonality may impact wastewater treatment efficacy for ARG removal, with distinct residue patterns observed across resistance genes. Thus, treatment strategies require seasonal optimization to ensure year-round consistent disinfection performance. Among the top 20 ARGs by relative abundance, *ermF, lnuH*, and *vanT* were more abundant in spring and summer effluent samples compared to influent samples, while their abundance decreased in autumn and winter effluent samples. The relative abundance of *mphG, fosA8,* and *soxR* increased in all effluent samples, and the ARGs that showed a significant decrease were primarily those involved in inactivating antibiotics, including those targeting aminoglycosides and penams. Continuous disinfection may selectively retain pathogens harboring resistant genes. These resistance genes can be vertically transmitted, thereby strengthening the resistance in their offspring. Different disinfectants have varying effects on different microbes, indirectly enriching certain resistance genes [46]. Shuai et al. collected public metagenomic data from 71 hospital wastewater samples across 18 hospitals and, using a novel assessment framework, identified 90 high-risk ARGs [47]. Our findings are consistent with those reported by Shuai et al., and include high-risk ARGs such as *tetA39*, *sul2*, *ermF*, *aph(6)-Id*, *blaOXA-58*, *aac3-lld*, *fosA8*, *blaNDM-1*, and *ermB*. These genes also align with the WHO high-priority list of clinically significant ARGs. Disinfectants containing benzalkonium chloride have been shown to selectively enrich multidrug ARGs, including *qacF_H, qacH_351*, *mdtg, msrE*, and *mtrD.* A study by Jia et al. [48,49] confirmed that disinfectants such as benzalkonium bromide, benzalkonium chloride, and polyhexamethylene guanidine hydrochloride promote the transformation and dissemination of plasmid-encoded ARGs by increasing membrane permeability [50]. Hospitals frequently use large amounts of various disinfectants, such as alcohol-based disinfectants, pasteurizing disinfectants, sodium hypochlorite, and potassium monopersulfate. Research has found that the detection rate of disinfectant resistance genes, such as *qacA/B*, is significantly higher in hospital-acquired MRSA isolates compared to community-acquired MRSA isolates. This difference is likely attributed to the intense disinfectant selection pressure these isolates experience within the hospital setting [51].

Currently, there is limited research on how Oxone affects the transformation and dissemination of ARGs. Analyzing the resistance mechanisms of ARGs, we find that antibiotic efflux and inactivation are the primary mechanisms in both influent and effluent samples. A single bacterium can carry multiple efflux pumps and selectively express key ones under antibiotic selection pressure. This allows for effective resistance to antibacterial drugs without significantly increasing the bacterial adaptation costs, thereby enabling pathogens to persist in both environmental and host settings [52]. Our study suggested that Oxone treatment altered the abundance of some microorganisms and ARGs. Despite the treatment, *qacG, adeF, tet39, msrE, mphG, mphE, ermF, aPH(6)-Id, sul2, and blaOXA-58* remain highly abundant. It is particularly noteworthy that the relative abundance of *soxR, fosA8, adeF,* and *VanO* increased compared to the influent after treatment. The incomplete inactivation of these ARGs may, to some extent, increase the risk of clinical treatment failure. *fosA8* is a gene that confers resistance to fosfomycin [53], a safe and effective drug used to treat infections caused by MDR bacteria. Three *fosA* genes, including *fosA3* and *fosA8*, were identified in clinical *E. coli* isolates from Canadian patients, with *fosA3* and *fosA8* confirmed as determinants of fosfomycin resistance [54]. The gene *sul2* has been reported to be widespread in rivers, soil, and wastewater treatment plant effluents [55,56]. *blaOXA-58* is a beta-lactamase enzyme found in *A. baumannii,* conferring resistance to penams or carbapenems. *A. baumannii* is recognized as a serious nosocomial pathogen and is commonly found in the natural environment [57]. Our study also detected two ARGs, *vanT* [58] and *vanO* [59], which interfere with peptidoglycan synthesis and reduce glycopeptide antibiotic binding affinity, thereby contributing to resistance against vancomycin and teicoplanin. The research findings suggested the potential risks associated with the widespread use of disinfectants and highlighted the necessity of optimizing hospital sewage treatment systems. These findings also indicate that different wastewater treatment processes have varying effects on the inactivation of diverse ARGs [39,60]. The ideal disinfection strategy has evolved from simply “killing bacteria” to a multi-objective process that requires simultaneously and efficiently inactivating ARB, degrading ARGs, and inhibiting horizontal gene transfer (HGT). UV disinfection can effectively inactivate ARB, but it struggles to completely degrade ARGs [61]. The UV dose required to inactivate ARGs is typically 10–20 times higher than that needed for ARB, leading to significantly increased costs [62]. Some ARGs (such as those in the *tet* family) exhibit strong resistance to UV and may undergo “photo-reactivation” after irradiation, which compromises treatment efficacy [63–65]. Furthermore, turbidity, organic matter, and nitrogen-containing compounds in wastewater absorb UV radiation, reducing its penetration and overall disinfection performance [64]. Therefore, UV is often combined with processes such as electrocoagulation to effectively remove ARGs [66]. Chlorination is widely used due to its low cost and broad application, primarily relying on oxidation to inactivate microorganisms [67]. However, it shows limited efficacy against certain ARB and may even promote the transfer of ARGs by damaging bacterial cell membranes, thereby exacerbating the spread of resistance [68]. It also produces approximately 600 types of harmful disinfection byproducts, posing risks to the environment and water quality. Its effectiveness is highly influenced by chlorine dosage, contact time, and water quality [69]. Advanced oxidation processes (AOPs, such as persulfate-based oxidation and ozonation) [11] and emerging technologies (e.g., plasma) [70] demonstrate significant advantages in removing ARGs and suppressing HGT. These include the absence of chemical byproducts, rapid action, no greenhouse gas emissions, and high efficiency in inactivating Gram-negative bacteria. Additionally, ozonation induces oxidative stress, enhancing its antibacterial effect [64,71]. However, these technologies also come with higher operational costs. Oxone, a peroxygen-based disinfectant used in this study, offers broad-spectrum antimicrobial activity, effectively breaks down organic matter, and produces no harmful byproducts [72]. Our results indicated that while Oxone treatment reduced the abundance of most bacteria and ARGs, its efficacy was considerably influenced by raw water temperature and water quality parameters. A study has demonstrated that, compared to direct chlorination disinfection processes, activated sludge treatment is more effective at eliminating ARGs [31]. Recent studies suggested that combined processes—such as integrating activated sludge with AOPs or UV—generally demonstrated superior efficacy in wastewater disinfection [67,73]. It is important to note that selecting the optimal disinfection method remains challenging and must be based on specific conditions, taking into account the advantages and limitations of each technology.

## 5 Limitations of the study

One limitation of our study is that we only detected resistance gene profiles in hospital wastewater. In future studies, we will correlate the resistance gene profiles of clinical bacterial isolates with those in hospital wastewater and systematically analyze the mechanisms underlying the progression and dissemination of resistance genes. By doing this, we hope to gain a deeper understanding of how these genes spread within and beyond hospital environments.

## 6 Conclusions

We characterized ARB and ARGs in the influent and effluent of a tertiary general hospital sewage treatment system in Nanchong City. After treatment, the total number of bacteria in hospital wastewater decreased. However, this wastewater treatment system exhibited a relatively poor germicidal effect on certain microorganisms, with a significant increase in relative abundance observed for the 3 phyla, 16 genera, and 21 species of microbes. Bacteria carrying ARGs were primarily affiliated with *Pseudomonadota* and *Bacillota*. A total of 354 ARGs were identified in the influent, and 331 in the effluent, with an overall decrease in abundance observed in the effluent. The relative abundance of *mphG, fosA8,* and *soxR* increased in all effluent samples, indicating that ARGs, ARBs, and other microorganisms can persist in hospital wastewater even after treatment, posing a significant risk to public health. This study emphasizes the importance of monitoring ARBs and ARGs in the influent and effluent of hospital sewage treatment systems, and it is crucial to adjust hospital wastewater disinfectants and optimize treatment processes to address antibiotic resistance.

## Supporting information

S1 Table Data preprocessing statistics for each sample. Sample: Sample Name; Insert Size(bp): Insert Fragment Length (default 350 bp library); Raw Data: Raw Sequencing Data; Clean Data: Filtered Valid Data; Clean_Q20: Percentage of bases in Clean Data with a sequencing error rate < 0.01 (quality score > 20); Clean_Q30: Percentage of bases in Clean Data with a sequencing error rate < 0.001 (quality score > 30); Clean_GC(%): GC Content in Clean Data; Effective(%): Percentage of Valid Data (Clean Data) relative to Raw Data (Raw Data).
(DOCX)

S2 Table Scaftigs for each sample. Sample: Indicates the samp; Total Len. (bp): Represents the total length of assembled Scaftigs; Num.: Indicates the total number of assembled Scaftigs; Average Len. (bp): Represents the average length of Scaftigs; N50 Len. (bp): Refers to the length value of Scaftigs when they are sorted by length and cumulatively summed from longest to shortest until the sum reaches 50% of the total length of all Scaftigs; N90 Len. (bp): Refers to the length value of Scaftigs when they are sorted by length and cumulatively summed from longest to shortest until the sum reaches 90% of the total length of all Scaftigs; Max Len: Indicates the length value of the longest assembled Scaftig.
(DOCX)

S3 Table Gene catalogue for each sample. ORFs NO.: Indicates the number of genes in the gene catalogue; integrity:none: Indicates the number and percentage of genes lacking both a start codon and a stop codon; integrity:end: Indicates the number and percentage of genes containing only a stop codon; integrity:start: Indicates the number and percentage of genes containing only a start codon; integrity:all: Indicates the percentage of complete genes (with both start and stop codons); Total Len.(Mbp): Represents the total length of genes in the gene catalogue, in million base pairs; Average Len.: Indicates the average length of genes in the gene catalogue; GC Percent: Represents the overall GC content of genes in the predicted gene catalogue.
(DOCX)

S4 Table Medical Wastewater Monitoring Data. COD limit: < 250 mg/L (Secondary Standard for effluent entering municipal networks). Residual chlorine limit: 2–8 mg/L (measured at the disinfection tank outlet). Standard ANOVA analysis revealed highly significant differences in mean values among groups, ***p < 0.001.
(DOCX)

S5 Table Alpha indexes statistics. Shannon: A higher Shannon index indicates greater community diversity. Simpson: The probability that two randomly sampled individuals belong to different species = 1-the probability that two randomly sampled individuals belong to the same species. The greater the Simpson index, the higher the community diversity. 1 At the phylum levels. 2 At the genus levels.
(DOCX)

S1 Fig Distribution of sequencing base mass (A) and sequencing base content (B) in each sample. (TIF)

S2 Fig Scaftigs Length distribution in each sample. The Frequence (#) represents the number of Scaftigs and the Percentage (%) represents the percentage of the number of Scaftigs.
(TIF)

S3 Fig Distribution of Gene catalogue Length in each sample. The Frequence (#) indicates the number of Gene catalogue and the Percentage (%) indicates the percentage of Gene catalogue number.
(TIF)

S4 Fig Metagenomic analysis of community structure in all samples visualized using KRONA. Circles represent different taxonomic levels (kingdom, phylum and class) in order from inside to outside, and the size of the sector represents the relative abundance of different species.
(TIF)

S5 Fig Dimensionality reduction analysis based on species abundance at various taxonomic levels: phylum (left), genera (middle), and species (right) levels. (A) PCA analysis, the x-axis represents the first principal component, with the percentage indicating its contribution to sample differences; the y-axis represents the second principal component, with the percentage indicating its contribution to sample differences. (B) PCoA analysis based on the Bray-Curtis distance, the x-axis represents one principal component and the y-axis represents another, with the percentage indicating its contribution to sample differences. (C) NMDS analysis, each point in the graph represents a sample, and the distance between the points indicates the degree of variation, with Stress less than 0.2 indicating reliability of the NMDS analysis. (D) Heatmap of species relative abundance clustering between groups at the phylum (left), genera (middle), and species (right).
(TIF)
