# Supplementary material for: Metagenomic analysis reveals the abundance changes of bacterial communities and antibiotic resistance genes in the influent and effluent of hospital wastewater
Source: PLoS One. 2025 Oct 31;20(10):e0335723. doi: 10.1371/journal.pone.0335723 (PMC12578235; doi:10.1371/journal.pone.0335723)
Supplement: S1 Table — Sample: Sample Name; Insert Size(bp): Insert Fragment Length (default 350 bp library); Raw Data: Raw Sequencing Data; Clean Data: Filtered Valid Data; Clean_Q20: Percentage of bases in Clean Data with a sequencing error rate < 0.01 (quality score > 20); Clean_Q30: Percentage of bases in Clean Data with a sequencing error rate < 0.001 (quality score > 30); Clean_GC(%): GC Content in Clean Data; Effective(%): Percentage of Valid Data (Clean Data) relative to Raw Data (Raw Data). (DOCX) [file pone.0335723.s001.docx]

| **Sample** | **InsertSize(bp)** | **RawData** | **CleanData** | **Clean_Q20** | **Clean_Q30** | **Clean_GC**  **(%)** | **Effective**  **(%)** |
| --- | --- | --- | --- | --- | --- | --- | --- |
| **SP.inf** | 350 | 6669.87 | 6656.25 | 97.44 | 92.89 | 53.45 | 99.796 |
| **SP.eff** | 350 | 7044.53 | 7018.41 | 96.78 | 91.56 | 58.15 | 99.629 |
| **SU.inf** | 350 | 6459.23 | 6446.47 | 97.19 | 92.31 | 44.97 | 99.803 |
| **SU.eff** | 350 | 6659.92 | 6646.2 | 96.84 | 91.66 | 50.06 | 99.794 |
| **FA.inf** | 350 | 6701.41 | 6682.11 | 97.31 | 92.69 | 53.13 | 99.712 |
| **FA.eff** | 350 | 6045.78 | 6027.64 | 97.43 | 92.97 | 57.84 | 99.7 |
| **WI.inf** | 350 | 6830.19 | 6800.25 | 97.12 | 92.28 | 52.6 | 99.562 |
| **WI.eff** | 350 | 6620.27 | 6603.05 | 97.42 | 92.99 | 59.04 | 99.74 |

**S1 Table. Data preprocessing statistics for each sample**

Sample: Sample Name

InsertSize(bp): Insert Fragment Length (default 350bp library)

RawData: Raw Sequencing Data

CleanData: Filtered Valid Data

Clean_Q20: Percentage of bases in CleanData with a sequencing error rate < 0.01 (quality score > 20)

Clean_Q30: Percentage of bases in CleanData with a sequencing error rate < 0.001 (quality score > 30)

Clean_GC(%): GC Content in CleanData

Effective(%): Percentage of Valid Data (CleanData) relative to Raw Data (RawData)
