# Supplementary material for: Metagenomic analysis reveals the abundance changes of bacterial communities and antibiotic resistance genes in the influent and effluent of hospital wastewater
Source: PLoS One. 2025 Oct 31;20(10):e0335723. doi: 10.1371/journal.pone.0335723 (PMC12578235; doi:10.1371/journal.pone.0335723)
Supplement: S2 Table — Sample: Indicates the samp; Total Len. (bp): Represents the total length of assembled Scaftigs; Num.: Indicates the total number of assembled Scaftigs; Average Len. (bp): Represents the average length of Scaftigs; N50 Len. (bp): Refers to the length value of Scaftigs when they are sorted by length and cumulatively summed from longest to shortest until the sum reaches 50% of the total length of all Scaftigs; N90 Len. (bp): Refers to the length value of Scaftigs when they are sorted by length and cumulatively summed from longest to shortest until the sum reaches 90% of the total length of all Scaftigs; Max Len: Indicates the length value of the longest assembled Scaftig. (DOCX) [file pone.0335723.s002.docx]

**S2 Table. Scaftigs for each sample.**

| **Sample** | **Total len.(bp)** | **Num.** | **Average len.(bp)** | **N50 Len.(bp)** | **N90 Len.(bp)** | **Max len.(bp)** |
| --- | --- | --- | --- | --- | --- | --- |
| **SP.inf** | 285,907,881 | 260,843 | 1,096.09 | 1,175 | 572 | 255,217 |
| **SP.eff** | 175,918,594 | 140,268 | 1,254.16 | 1,417 | 585 | 126,187 |
| **SU.inf** | 199,427,960 | 173,573 | 1,148.96 | 1,281 | 577 | 90,384 |
| **SU.eff** | 136,587,332 | 99,725 | 1,369.64 | 1,718 | 592 | 159,621 |
| **FA.inf** | 264,822,482 | 244,223 | 1,084.35 | 1,150 | 572 | 126,914 |
| **FA.eff** | 128,845,738 | 94,637 | 1,361.47 | 1,741 | 593 | 155,620 |
| **WI.inf** | 249,163,814 | 216,678 | 1,149.93 | 1,271 | 578 | 96,379 |
| **WI.eff** | 144,561,206 | 128,416 | 1,125.73 | 1,229 | 583 | 113,994 |

Sample: Indicates the sample name.

Total Len. (bp): Represents the total length of assembled Scaftigs.

Num.: Indicates the total number of assembled Scaftigs.

Average Len. (bp): Represents the average length of Scaftigs.

N50 Len. (bp): Refers to the length value of Scaftigs when they are sorted by length and cumulatively summed from longest to shortest until the sum reaches 50% of the total length of all Scaftigs.

N90 Len. (bp): Refers to the length value of Scaftigs when they are sorted by length and cumulatively summed from longest to shortest until the sum reaches 90% of the total length of all Scaftigs.

Max Len: Indicates the length value of the longest assembled Scaftig.
