# Supplementary material for: Metagenomic analysis reveals the abundance changes of bacterial communities and antibiotic resistance genes in the influent and effluent of hospital wastewater
Source: PLoS One. 2025 Oct 31;20(10):e0335723. doi: 10.1371/journal.pone.0335723 (PMC12578235; doi:10.1371/journal.pone.0335723)
Supplement: S3 Table — ORFs NO.: Indicates the number of genes in the gene catalogue; integrity:none: Indicates the number and percentage of genes lacking both a start codon and a stop codon; integrity:end: Indicates the number and percentage of genes containing only a stop codon; integrity:start: Indicates the number and percentage of genes containing only a start codon; integrity:all: Indicates the percentage of complete genes (with both start and stop codons); Total Len.(Mbp): Represents the total length of genes in the gene catalogue, in million base pairs; Average Len.: Indicates the average length of genes in the gene catalogue; GC Percent: Represents the overall GC content of genes in the predicted gene catalogue. (DOCX) [file pone.0335723.s003.docx]

| **Sample** | **ORFs NO** | **integrity:none** | **integrity:end** | **integrity:start** | **integrity:all** | **Total length** | **Average length** | **GC percent** |
| --- | --- | --- | --- | --- | --- | --- | --- | --- |
| **FA.eff** | 187,100 | 20,368(10.89%) | 40,749(21.78%) | 51,602(27.58%) | 74,381(39.75%) | 115.38 | 616.69 | 59.31 |
| **FA.inf** | 414,002 | 49,368(11.92%) | 104,421(25.22%) | 131,866(31.85%) | 128,347(31%) | 232.58 | 561.77 | 54.42 |
| **SP.eff** | 266,922 | 29,563(11.08%) | 60,885(22.81%) | 77,940(29.2%) | 98,534(36.91%) | 157.35 | 589.49 | 59.79 |
| **SP.inf** | 444,977 | 52,758(11.86%) | 112,173(25.21%) | 139,174(31.28%) | 140,872(31.66%) | 251.36 | 564.89 | 54.92 |
| **SU.eff** | 197,774 | 20,579(10.41%) | 42,856(21.67%) | 54,128(27.37%) | 80,211(40.56%) | 120.76 | 610.6 | 58.04 |
| **SU.inf** | 305,189 | 33,142(10.86%) | 74,949(24.56%) | 95,920(31.43%) | 101,178(33.15%) | 174.68 | 572.37 | 50.13 |
| **WI.eff** | 226,625 | 26,737(11.8%) | 56,327(24.85%) | 73,332(32.36%) | 70,229(30.99%) | 129.75 | 572.53 | 60.6 |
| **WI.inf** | 378,846 | 43,705(11.54%) | 91,770(24.22%) | 118,848(31.37%) | 124,523(32.87%) | 220.49 | 582 | 53.75 |

**S3 Table. Gene catalogue for each sample.**

ORFs NO. : Indicates the number of genes in the gene catalogue

integrity:none : Indicates the number and percentage of genes lacking both a start codon and a stop codon

integrity:end : Indicates the number and percentage of genes containing only a stop codon

integrity:start : Indicates the number and percentage of genes containing only a start codon

integrity:all : Indicates the percentage of complete genes (with both start and stop codons)

Total Len.(Mbp) : Represents the total length of genes in the gene catalogue, in million base pairs

Average Len. : Indicates the average length of genes in the gene catalogue

GC Percent : Represents the overall GC content of genes in the predicted gene catalogue.
