# Supplementary material for: Metagenomic analysis reveals the abundance changes of bacterial communities and antibiotic resistance genes in the influent and effluent of hospital wastewater
Source: PLoS One. 2025 Oct 31;20(10):e0335723. doi: 10.1371/journal.pone.0335723 (PMC12578235; doi:10.1371/journal.pone.0335723)
Supplement: S4 Table — COD limit: < 250 mg/L (Secondary Standard for effluent entering municipal networks). Residual chlorine limit: 2–8 mg/L (measured at the disinfection tank outlet). Standard ANOVA analysis revealed highly significant differences in mean values among groups, ***p < 0.001. (DOCX) [file pone.0335723.s004.docx]

**S4 Table. Medical Wastewater Monitoring Data.**

| **Season** | **COD (mg/L)** | **Residual chlorine level (mg/L)** | **Outlet water temperature（℃）** |
| --- | --- | --- | --- |
| **March** | 60.21±13.74 | 2.64±0.26 | 18.53±0.75*** |
| **July** | 77.52±13.54 | 3.36±1.74 | 28.03±1.23*** |
| **October** | 60.53±45.16 | 4.34±1.54 | 24.14±2.13*** |
| **December** | 117.96±35.27 | 2.32±0.37 | 16.3±4.15*** |

COD limit: < 250 mg/L (Secondary Standard for effluent entering municipal networks).

Residual chlorine limit: 2–8 mg/L (measured at the disinfection tank outlet).

Standard ANOVA analysis revealed highly significant differences in mean values among groups, ***p<0.001.
