# Supplementary material for: Metagenomic analysis reveals the abundance changes of bacterial communities and antibiotic resistance genes in the influent and effluent of hospital wastewater
Source: PLoS One. 2025 Oct 31;20(10):e0335723. doi: 10.1371/journal.pone.0335723 (PMC12578235; doi:10.1371/journal.pone.0335723)
Supplement: S5 Table — Shannon: A higher Shannon index indicates greater community diversity. Simpson: The probability that two randomly sampled individuals belong to different species = 1-the probability that two randomly sampled individuals belong to the same species. The greater the Simpson index, the higher the community diversity. 1 At the phylum levels. 2 At the genus levels. (DOCX) [file pone.0335723.s005.docx]

**S5 Table. Alpha indexes statistics**

| **Season** | **Shannon^1^** | **Simpson^1^** | **Shannon^2^** | **Simpson^2^** |
| --- | --- | --- | --- | --- |
| **SP.inf** | 0.99362 | 0.31994 | 4.05574 | 0.89030 |
| **SP.eff** | 0.85243 | 0.26559 | 3.22241 | 0.76717 |
| **SU.inf** | 1.62239 | 0.59252 | 3.52033 | 0.77963 |
| **SU.eff** | 1.25427 | 0.45667 | 3.22132 | 0.81191 |
| **FA.inf** | 0.88458 | 0.27195 | 3.39383 | 0.79367 |
| **FA.eff** | 0.53306 | 0.12536 | 1.81118 | 0.36911 |
| **WI.inf** | 1.41577 | 0.46664 | 4.02225 | 0.86242 |
| **WI.eff** | 0.37283 | 0.08999 | 1.59147 | 0.30165 |

Shannon: A higher Shannon index indicates greater community diversity. Simpson: The probability that two randomly sampled individuals belong to different species = 1-the probability that two randomly sampled individuals belong to the same species. The greater the Simpson index, the higher the community diversity.

1 At the phylum levels.

2 At the genus levels.
