# Supplementary figures and images for: Metagenomic analysis reveals the abundance changes of bacterial communities and antibiotic resistance genes in the influent and effluent of hospital wastewater
Source: PLoS One. 2025 Oct 31;20(10):e0335723. doi: 10.1371/journal.pone.0335723 (PMC12578235; doi:10.1371/journal.pone.0335723)

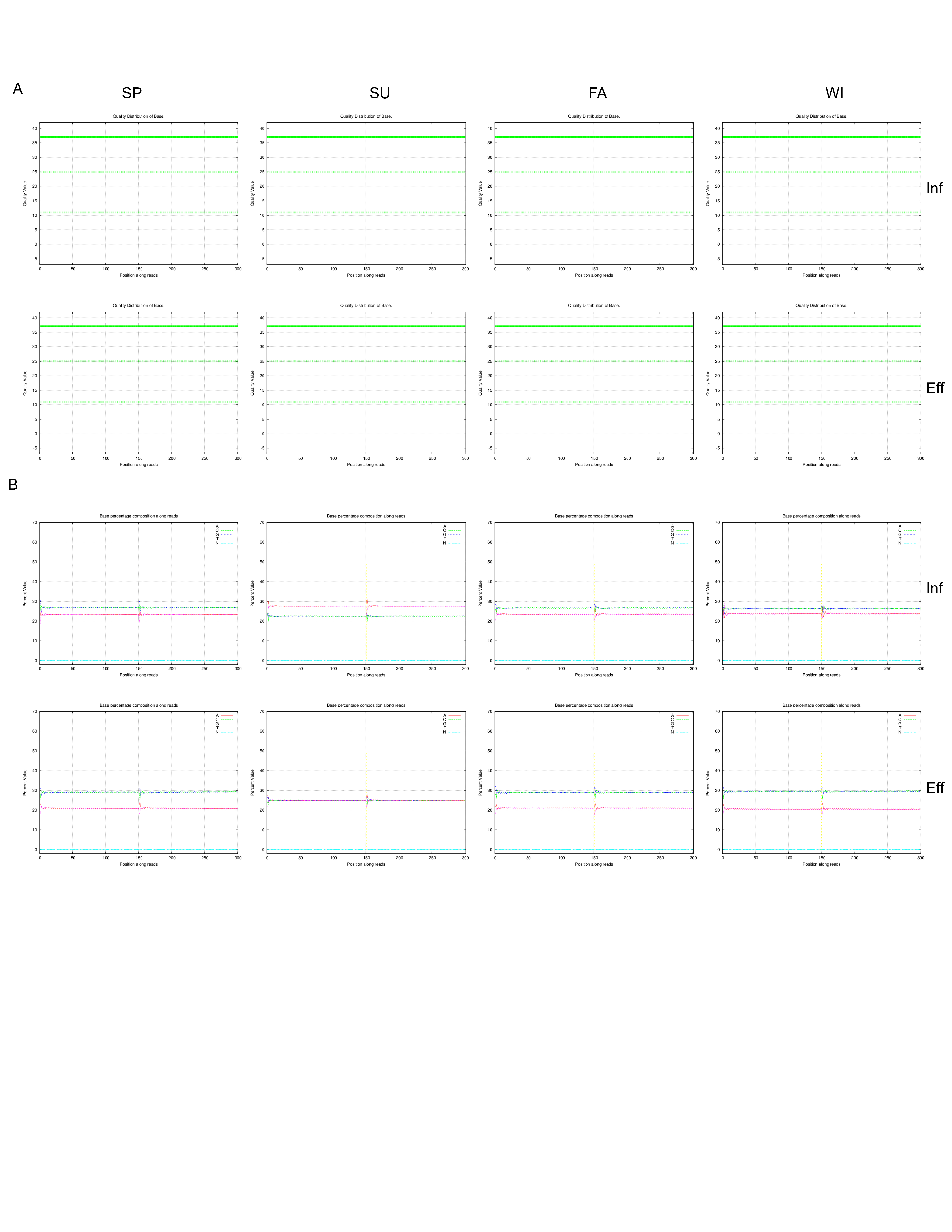

Supplement: S1 Fig — (TIF) [file pone.0335723.s006.tif]

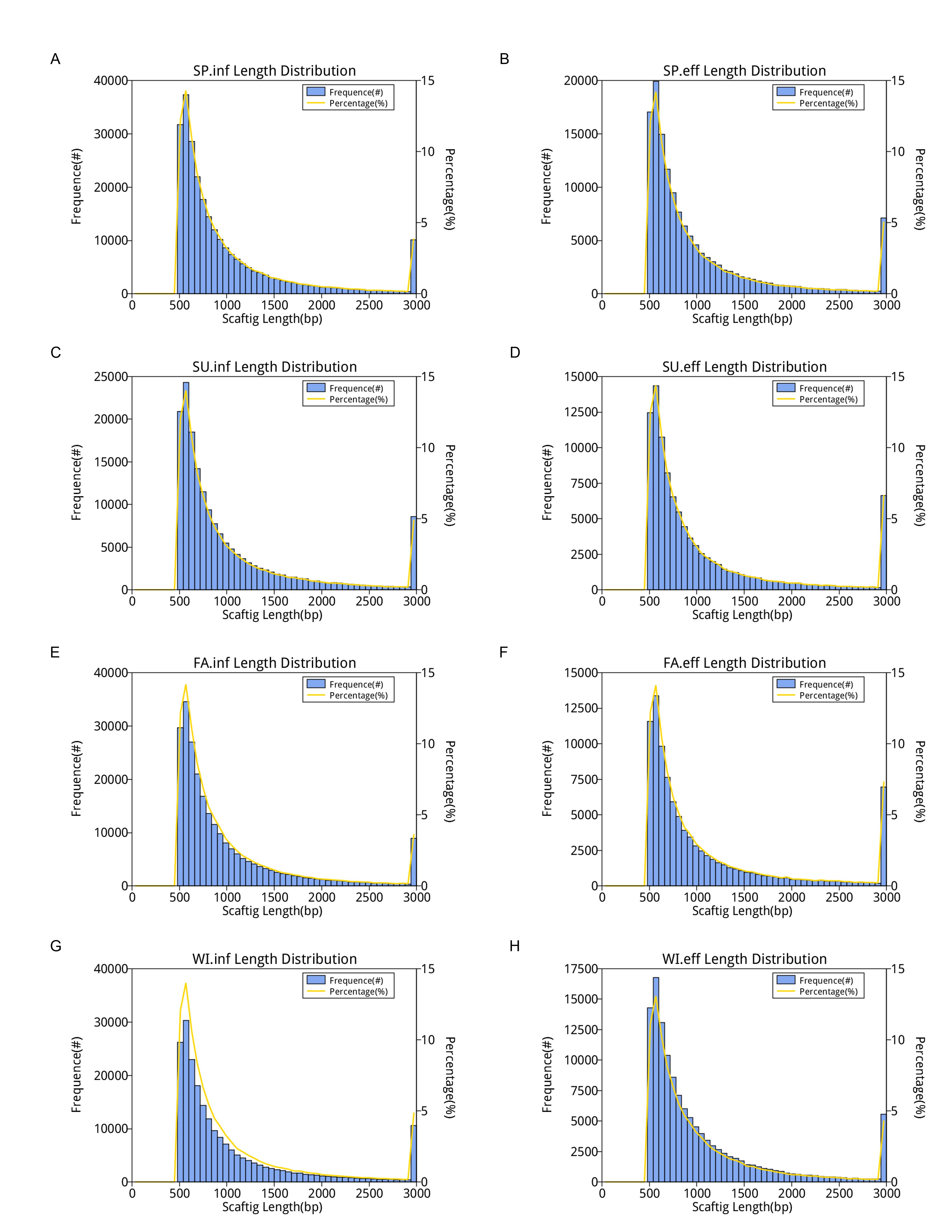

Supplement: S2 Fig — The Frequence (#) represents the number of Scaftigs and the Percentage (%) represents the percentage of the number of Scaftigs. (TIF) [file pone.0335723.s007.tif]

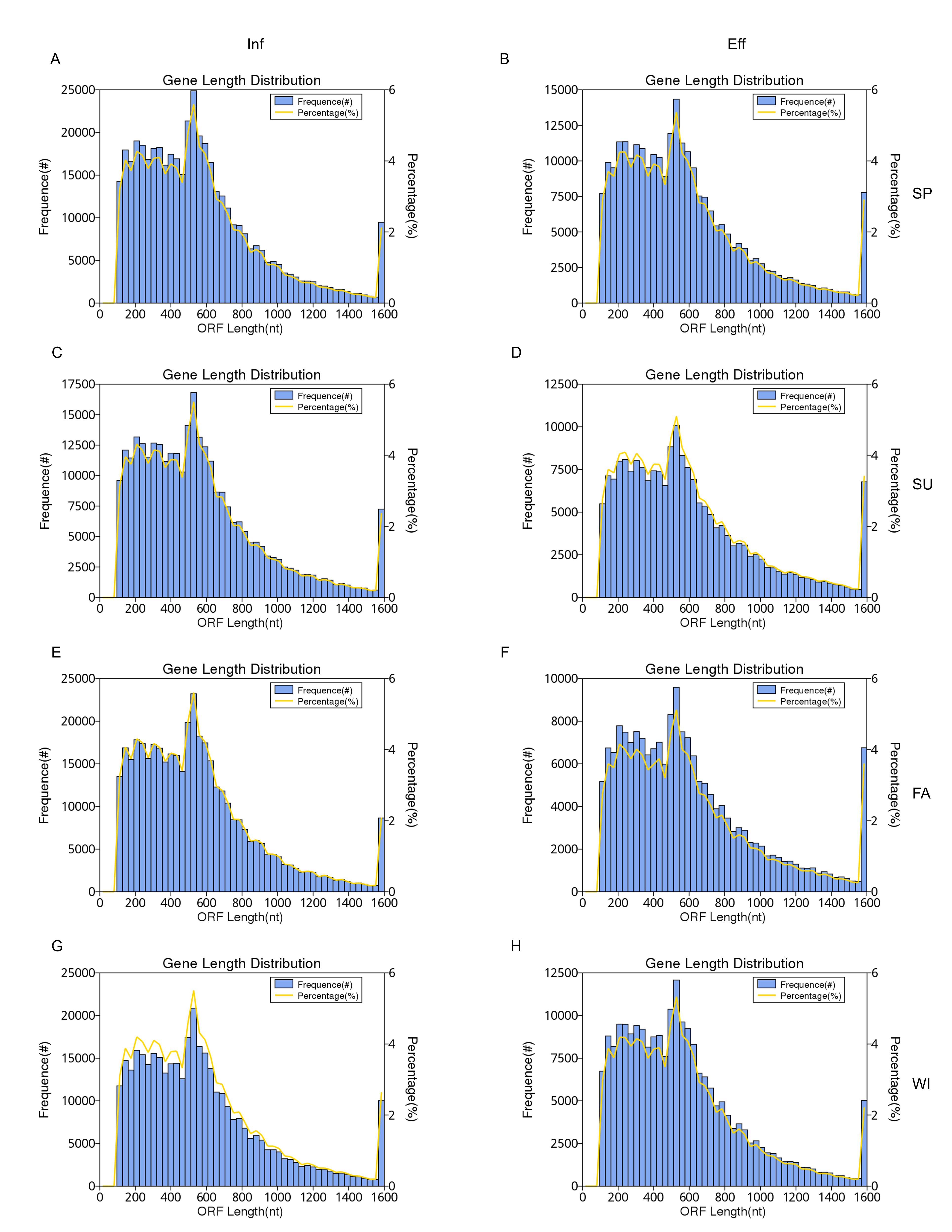

Supplement: S3 Fig — The Frequence (#) indicates the number of Gene catalogue and the Percentage (%) indicates the percentage of Gene catalogue number. (TIF) [file pone.0335723.s008.tif]

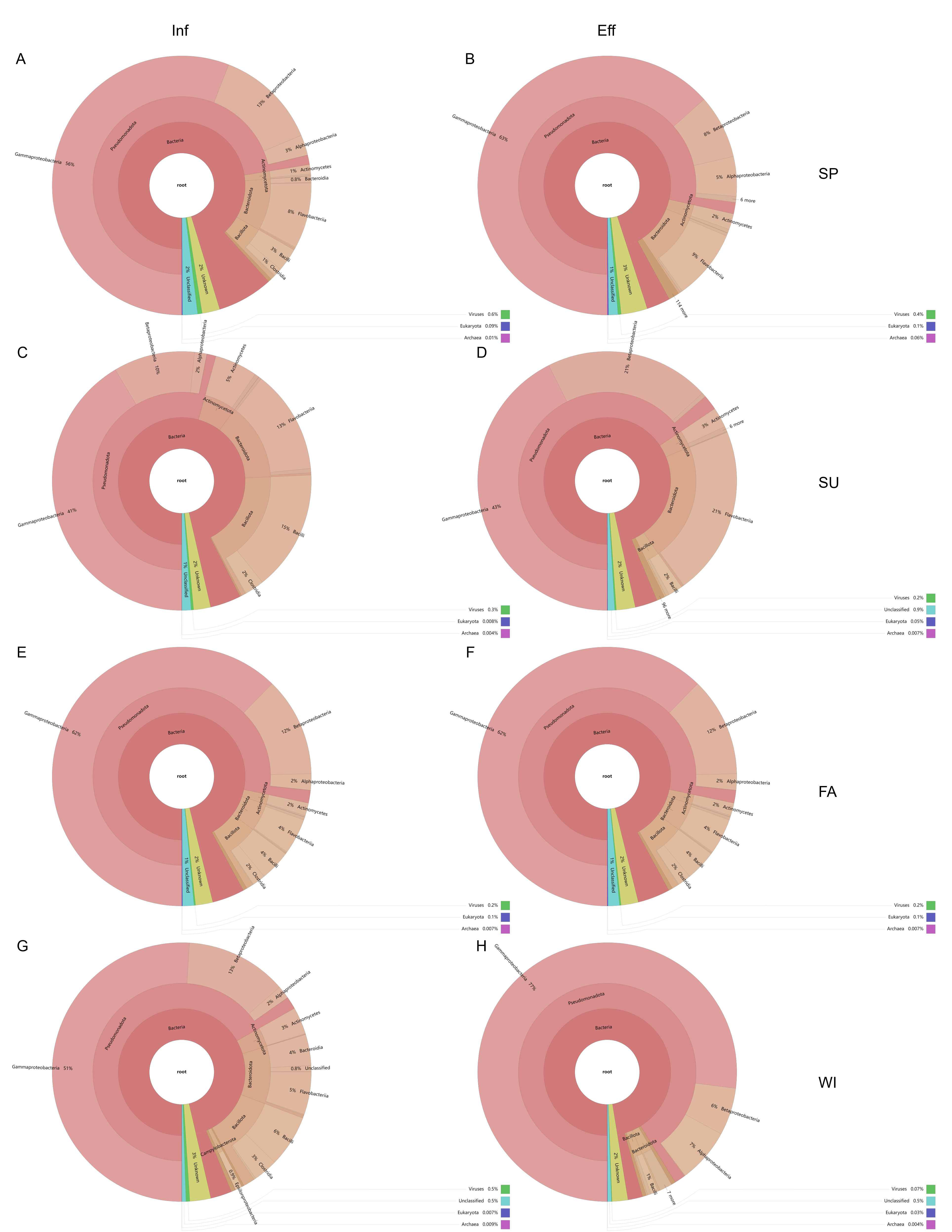

Supplement: S4 Fig — Circles represent different taxonomic levels (kingdom, phylum and class) in order from inside to outside, and the size of the sector represents the relative abundance of different species. (TIF) [file pone.0335723.s009.tif]

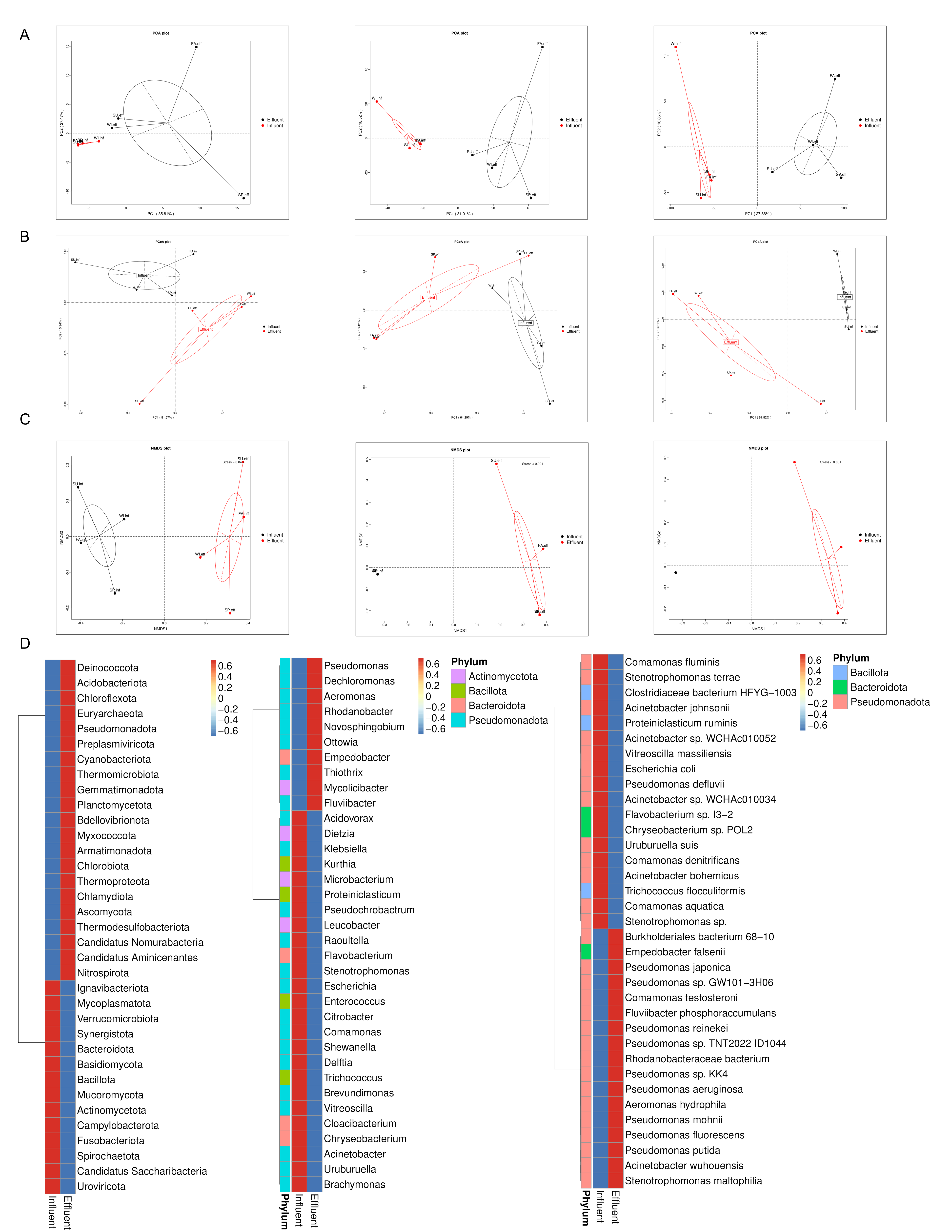

Supplement: S5 Fig — (A) PCA analysis, the x-axis represents the first principal component, with the percentage indicating its contribution to sample differences; the y-axis represents the second principal component, with the percentage indicating its contribution to sample differences. (B) PCoA analysis based on the Bray-Curtis distance, the x-axis represents one principal component and the y-axis represents another, with the percentage indicating its contribution to sample differences. (C) NMDS analysis, each point in the graph represents a sample, and the distance between the points indicates the degree of variation, with Stress less than 0.2 indicating reliability of the NMDS analysis. (D) Heatmap of species relative abundance clustering between groups at the phylum (left), genera (middle), and species (right). (TIF) [file pone.0335723.s010.tif]
